# Cascaded All-Fiber Gas Raman Laser Oscillator in Deuterium-Filled Hollow-Core Photonic Crystal Fibers

**DOI:** 10.3390/nano14080661

**Published:** 2024-04-11

**Authors:** Hao Li, Wenxi Pei, Xuanxi Li, Luohao Lei, Jing Shi, Zhiyue Zhou, Zefeng Wang

**Affiliations:** 1 College of Advanced Interdisciplinary Studies, National University of Defense Technology, Changsha 410073, China; lihao18c@nudt.edu.cn (H.L.); peiwenxi@nudt.edu.cn (W.P.); lixuanxi113@nudt.edu.cn (X.L.); leiluohao18@nudt.edu.cn (L.L.); shijing18@nudt.edu.cn (J.S.); zhouzhiyue12@nudt.edu.cn (Z.Z.); 2 Nanhu Laser Laboratory, National University of Defense Technology, Changsha 410073, China; 3 State Key Laboratory of Pulsed Power Laser Technology, Changsha 410073, China

**Keywords:** laser–matter interaction, hollow-core fiber, fiber laser

## Abstract

Hollow-core photonic crystal fibers (HC-PCFs) provide an ideal transmission medium and experimental platform for laser–matter interaction. Here, we report a cascaded all-fiber gas Raman laser based on deuterium (D_2_)-filled HC-PCFs. D_2_ is sealed into a gas cavity formed by a 49 m-long HC-PCF and solid-core fibers, and two homemade fiber Bragg gratings (FBGs) with the Raman and pump wavelength, respectively, are further introduced. When pumped by a pulsed fiber amplifier at 1540 nm, the pure rotational stimulated Raman scattering of D_2_ occurs inside the cavity. The first-order Raman laser at 1645 nm can be obtained, realizing a maximum power of ~0.8 W. An all-fiber cascaded gas Raman laser oscillator is achieved by adding another 1645 nm high-reflectivity FBG at the output end of the cavity, reducing the peak power of the cascaded Raman threshold by 11.4%. The maximum cascaded Raman power of ~0.5 W is obtained when the pump source is at its maximum, and the corresponding conversion efficiency inside the cavity is 21.4%, which is 1.8 times that of the previous configuration. Moreover, the characteristics of the second-order Raman lasers at 1695 nm and 1730 nm are also studied thoroughly. This work provides a significant method for realizing all-fiber cascaded gas Raman lasers, which is beneficial for expanding the output wavelength of fiber gas lasers with a good stability and compactivity.

## 1. Introduction

As a new type of optical fiber, HC-PCFs provide an ideal transmission medium and experimental platform for laser–matter interaction and have received extensive attention and application in many fields, such as sensing, energy transmission, and communication [1,2,3,4,5]. Unlike traditional optical fibers, HC-PCFs have a hollow core, and the cladding is composed of periodically arranged microstructures with nanometer thickness. Therefore, HC-PCFs can confine the laser light to a hollow fiber core for long-distance transmission, which greatly enhances the interaction between the laser and matter. In 2002, Benabid et al. first reported a gas Raman laser based on HC-PCFs, which reduced the threshold of the stimulated Raman scattering (SRS) of hydrogen by two orders of magnitude based on hydrogen-filled HC-PCFs [6]. Subsequently, fiber gas Raman lasers (FGRLs) have received more and more attention and research [7,8,9,10,11]. This novel fiber laser not only has the advantages of a good beam quality, stability, and heat dissipation like traditional fiber lasers [12], but also combines the convenience of gas Raman lasers in wavelength conversion [13,14,15], which has great potential in expanding the laser wavelength. Up until now, FGRLs have realized a laser output in various wavelengths, from the ultraviolet to mid-infrared band [16,17,18,19,20,21,22,23,24], and enrich the wavelength of fiber lasers. However, almost all of these works only obtain the direct output of a first-order Raman laser. If the first-order Raman laser can work as the pump laser and induce cascaded Raman conversion, cascaded fiber Raman lasers with longer wavelengths can be realized, which is quite profound in enriching and expanding the wavelength.

In 2010, a cascaded FGRL on HC-PCFs has been reported by Beaudou et al. [25]. In this work, the pump wavelength is 1064 nm, and the highest fifth-order Raman line is obtained at 1549 nm. However, the HC-PCF is tapered to expand the transmission bandwidth to transmit high-order Raman lines, so this operation is quite complex and not universal. With the development of anti-resonance hollow-core fibers (AR-HCF) [26,27], cascaded gas Raman has shown more advantages, and cascaded Raman light output at mid-infrared wavelengths can be achieved by using pump sources of common wavelengths. In 2018, Li et al. demonstrated a cascaded 2.8 μm FGRL based on two stages of methane-filled hollow-core fibers (HCFs) [28]. The first stage is pumped by a commercial 1064.6 nm laser; then, the obtained first-order Raman laser at 1543.9 nm is coupled into the second stage, realizing a cascaded Raman laser output. However, the multistage set makes the experimental setup bulky and complex, so realizing cascaded Raman conversion in a single HCF is another feasible choice. In the same year, the cascaded FGRL based on a single gas-filled AR-HCF was also reported [20,21]. Cao et al. [20] used a 1064 nm laser to pump a methane-filled AR-HCF to achieve a cascaded gas Raman laser output at 2.8 μm. Moreover, Gladyshev et al. [21] utilized the cascaded rotational or vibrational Raman conversion in a H_2_/D_2_-filled AR-HCF pumped by a 1558 nm laser, realizing a cascaded Raman laser at 3.3 μm and 3.5 μm. In 2023, Lanari et al. reported a 2.58 μm cascaded FGRL based on a methane-filled AR-HCF [29]. The pump wavelength is 1030 nm, and the first- and second-order Raman wavelength is about 1472 nm and 2580 nm, respectively. However, in the works above [20,21,25,29], all cascaded FGRLs couple a pump laser into the gas-filled HCF through free space, giving an unstable and bulky system. Therefore, the motivation of this work is to realize a cascaded FGRL with an all-fiber structure, which will greatly enhance the stability and compactness of the system.

Furthermore, most of the output wavelengths of cascaded FGRLs are concentrated in the mid-infrared band, and other special bands, such as the 1.7 μm band, have not been studied. In fact, due to the unique spectral characteristics of the 1.7 μm band, 1.7 μm fiber lasers have a wide range of applications in the fields of biological imaging, gas detection, material processing, mid-infrared laser generation, and so on [30,31,32,33]. In terms of biological imaging and gas detection, 1.7 μm pulsed lasers are used to achieve a higher sensitivity and deeper penetration and detection depth [32,33]. However, because thulium-doped fibers demonstrate strong reabsorption in the 1.7 μm band [34], and the fabrication technology of bismuth-doped fibers is immature [35], there are great challenges in obtaining 1.7 μm band lasers in traditional solid-core fiber lasers. Therefore, the realization of the 1.7 μm laser output by the cascaded FGRL is also of great significance in enriching the output wavelength of fiber lasers and promoting the practical application of cascaded FGRLs.

Here, we have demonstrated a 1.7 μm all-fiber cascaded FGRL based on a D_2_-filled HC-PCF. An all-fiber gas cavity is fabricated by splicing solid-core fibers with 49 m HC-PCFs filled by ~24.5 bar of deuterium, and the homemade FBGs with a high reflectivity are introduced at the input and output ends. When pumped by the laser at 1540 nm, the pure rotational SRS of D_2_ molecules occurs inside the cavity and the first-order Raman power of ~0.8 W at 1645 nm is obtained. By introducing another FBG at the first-order Raman wavelength, an all-fiber cascaded FGRL oscillator is achieved. When the pump source is at the maximum power, the cascaded second-order Raman power of ~0.5 W is realized, containing the lasers at 1695 nm and 1730 nm. The Raman threshold of the cascaded Raman laser is reduced dramatically and the cascaded Raman power is increased by 1.8 times that of the single-pass configuration in this work. This present result is another exploration of the FGRLs and offers a feasible way for realizing all-fiber cascaded Raman lasers based on HCFs.

## 2. Experimental Setup

**Figure 1:**
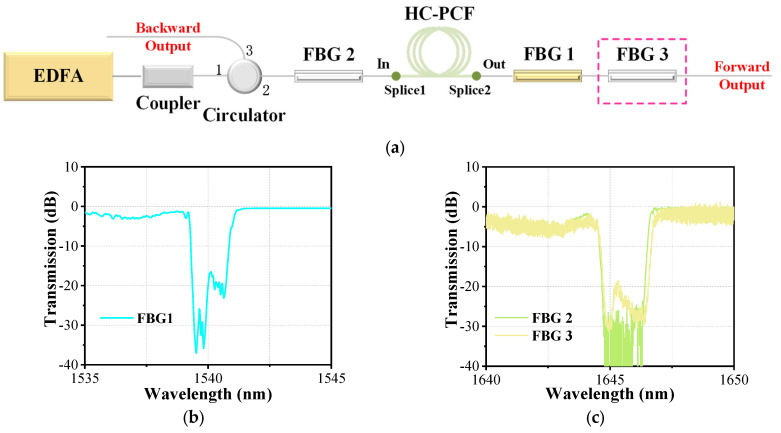
(**a**) The schematic diagram of experimental setup; (**b**) the transmission spectrum of the FBG1; and (**c**) the transmission spectrum of the FBG2 and FBG3.

Figure 1a shows the schematic diagram of the experimental setup. A homemade erbium-doped fiber amplifier (EDFA) emitting a 1540 nm narrow-linewidth laser works as the pump source. The repetition frequency is adjustable, and the pulse width can be set from 10 ns to 30 ns. The pump source shows similar power characteristics when pulse properties change. Its maximum average output power of ~6.8 W can be obtained and the fluctuation ratio is less than ~0.6% in 7000 s. A fiber coupler is spliced to the pigtail of the pump source directly, which can monitor the real output power (the measured coupling ratio is ~99:1). Then, the main output end of the coupler is spliced to the port1 of a circulator. Due to the same fiber type (SMF-28e, Corning Incorporated, Corning, New York, NY, USA), the splicing loss can be ignored, but the insert loss of the circulator is nonnegligible, and its value is ~0.7 dB. The medium that provides the place for the interaction between gas and laser is a 49 m-long HC-PCF, which is the same as the fiber used in our previous work [36]. Its core diameter and the mode field diameter are ~10 μm and ~9 μm, respectively. In the range of 1500–1700 nm, the transmission loss is less than 0.08 dB/m. To fabricate the all-fiber gas cavity, we fusion-spliced one end of the HC-PCFs with a single-mode solid-core fiber (SMF-28e, which has a relatively close mode field diameter of ~10 μm) as the input end of the cavity. The splicing loss of the splice is estimated to be ~1.4 dB. Then, we sealed the other end of the HC-PCF into a homemade gas cell and vacuumed it with a customized vacuum pump. After that, the HC-PCF is filled with high-pressure D_2_ and stands for more than 10 h. When the gas inside the hollow core is balanced, take it out of the gas cell and splice it with another solid-core fiber quickly, which works as the output end of the gas cavity. The splicing loss is estimated to be 1.9 dB. Due to the HC-PCF being filled with gas, the parameters of the fusion splice should be adjusted slightly to protect the hollow core from collapsing. By calculating the amount of gas leakage [37], the final gas pressure inside the gas cavity is estimated to be ~24.5 bar. It should be noted that the all-fiber gas cavity is fabricated by directly fusion-splicing a HC-PCF and solid-core fibers, which is only suitable for solid-core fibers and HC-PCFs with similar diameters of the core and the mode field. If there is a large difference in the core and mode field diameters between the solid-core fiber and the HC-PCFs, it is necessary to taper or expand the core of the solid-core fiber to match the core and mode field diameters of the two, to reduce the splice loss [38,39]. Moreover, the all-fiber gas cavity has good thermal stability, and only when the temperature is high enough to cause the microstructure inside the HC-PCFs to expand and deform (which may be as high as several hundred degrees Celsius in quartz HC-PCFs) could the loss characteristics of the fusion point and the transmission characteristics of the all-fiber gas cavity be affected. However, the all-fiber gas cavity is sensitive to mechanical stress, especially transverse stress, which may cause damage to fusion points and the deformation of the microstructure inside HC-PCFs. 

For our experiment, we obtained the laser output by pumping different combinations of the D_2_-filled all-fiber gas cavity and FBGs. Firstly, we fusion-spliced the input end of the gas cavity with a 1645 nm FBG (marked as FBG2 in Figure 1a), which had been spliced with the circulator port3, and then added a 1540 nm FBG (marked as FBG1) at the output end and measured its output characteristics. We define it as Setup 1. Subsequently, we added another FBG at 1645 nm (marked as FBG3) at the output end of FBG1, realizing a cascaded FGRL oscillator, which is named Setup 2. These FBGs were inscribed in single-mode fibers (SMF-28e, Corning Incorporated, Corning, New York, NY, USA) by using ultraviolet laser phase mask technology, and their transmission spectra are shown in Figure 1b,c. The center wavelength of the FBG1 transmission spectrum is 1540 nm with a depth of greater than 20 dB, corresponding to a reflectivity of greater than 99%. The FBG2 and FBG3 have the same center wavelength of 1645 nm with a depth of greater than 20 dB, and the corresponding reflectivity is also greater than 99%. Moreover, the bandwidth of these FBGs is sufficient to cover the pump line and Raman line because the linewidth of the pump light and Raman light is very narrow.

## 3. Results and Discussion

### 3.1. Output Spectrum

**Figure 2:**
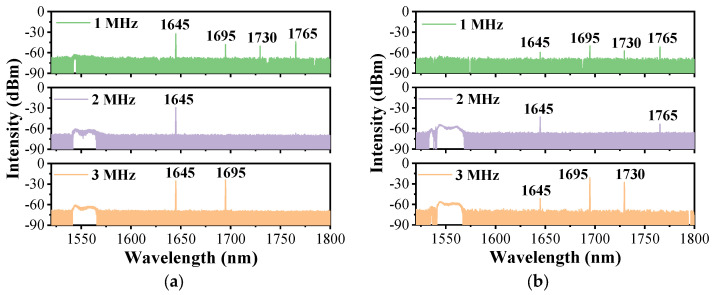
Measured forward spectrum of (**a**) Setup 1 and (**b**) Setup 2 at the maximum pump power with the different repetition frequencies.

Figure 2a shows the output spectrum of Setup 1 with the different repetition frequencies when the pump power is at its maximum. Considering the influence of the pulse peak power on the Raman threshold of the D_2_ gas and the nonlinear effect in the solid-core fiber, the pulse width is set to 20 ns. It can be seen that, due to the reflection at 1540 nm offered by FBG1, no pump line is detected in all three cases. The added FBG1 at the pump wavelength increases the interaction distance between the gas and lasers and improves the utilization of the pump light. For the case with a repetition frequency of 1 MHz, as the peak power of the pulse is sufficiently high, more than one Raman line is generated and the cascaded Raman conversion occurs apparently. The pump light is converted into the first-order Raman light at 1645 nm with a Raman shift of ~414 cm^−1^. The lines at 1695 nm, 1730 nm, and 1765 nm are cascaded second-order Raman lines that all converted from the first-order Raman line at 1645 nm, and the corresponding Raman shifts are ~179 cm^−1^, ~298 cm^−1^, and ~414 cm^−1^, respectively. All the cascaded Raman lines have a similar and weak intensity, which is mainly due to the complicated conversion and the dispersed energy of the second-order Raman pulse. When the repetition frequency is adjusted to 2 MHz, the peak power of the pump pulse is reduced by two times, and only first-order Raman conversion occurs. The absence of the other Raman lines implies the cascaded Raman conversion is inadequate. For the case with a repetition frequency of 3 MHz, the peak power of the pump pulse keeps decreasing, but there are still second-order Raman lines at 1695 nm and 1730 nm, except for the line at 1765 nm. This is mainly because the positive feedback provided by the gas cavity makes the pulses overlap, reducing the Raman threshold power of these two lines [23], but the peak power of the pump pulse is still lower than the threshold peak power of the Raman line at 1765 nm, and the transmission loss at this wavelength is also much higher. 

Figure 2b presents the spectral characteristics of the FGRL with Setup 2. When pumped at the maximum power, its output spectrum shows similar compositions, but the intensity is quite different. For the case with a repetition frequency of 1 MHz, the additionally added FBG3 reflects the laser at 1645 nm back into the cavity; thus, the line at this wavelength becomes weaker. The cascaded Raman conversion still occurs and their intensity resembles that of Setup 1. When the repetition frequency increases to 2 MHz, besides a weak 1645 nm line, there is another weak line at 1765 nm, which corresponds to the strongest Raman gain among the other high-order Raman lines [40]. It indicates that the added FBG3 promotes the cascaded Raman conversion. For the case with a repetition frequency of 3 MHz, compared to the spectrum in Figure 2a, the main difference between them is only the intensity at 1645 nm, and the lines at 1695 nm and 1730 nm are unstable and changeable in both of them; we will discuss this in Section 3.3.

### 3.2. Output Pulse Shape

**Figure 3:**
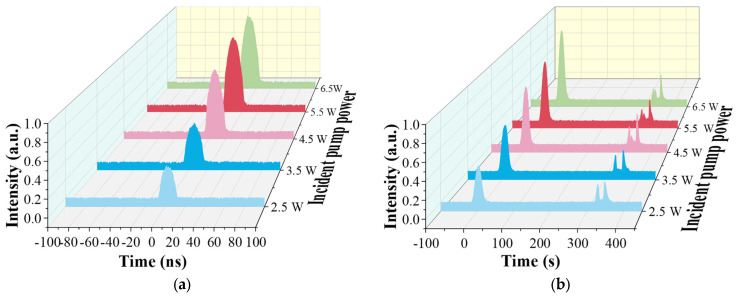
Measured (**a**) forward and (**b**) backward pulse shapes at the maximum pump power when only FBG1 is added.

By using bandpass filters with a different transmittance, we can separate the pump pulse and the Raman pulse effectively, and then delve into its temporal characteristic. To explore the temporal characteristics of the first-order Raman conversion, we set the pulse width to 20 ns with a repetition frequency of 2 MHz in Setup 1, and measured the evolution of pulse shapes with the incident pump power (the actual output power at the circulator port2), as shown in Figure 3. For the forward pulse in Figure 3a, there is only the first-order Raman pulse at 1645 nm detected. These pulses show a good Gaussian shape and become wider with the increasing of the incident pump power, which is because more and more energy of the pump pulse beyond the Raman threshold participates in Raman conversion. The residual pump pulse of the forward output is reflected into the cavity by the FBG1 and the second-order Raman pulse is too weak to be measured. Figure 3b shows the pulse shapes of the backward output. The pulse detected earlier is the pump pulse, which is mainly from the forward pump pulse’s reflection at splice 1. Another pulse detected later is the backward pump pulse that is reflected by the FBG1, and the dip in the profile of these pulses is caused by the Raman conversion. Moreover, we also detected the backward Raman pulse at 1645 nm, and its evolution is similar to that of the forward Raman pulse, so we omit its analysis.

**Figure 4:**
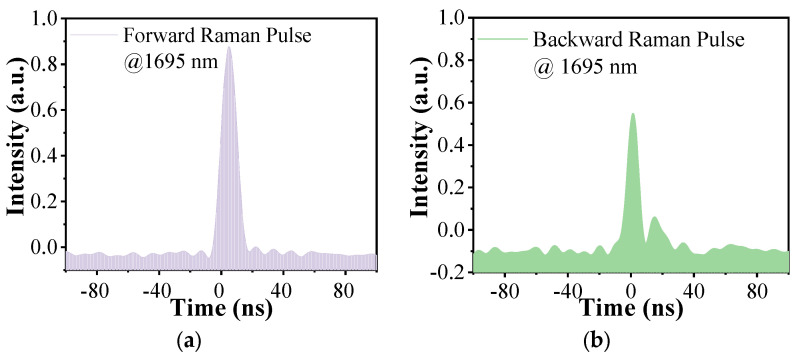
Measured (**a**) forward and (**b**) backward high-order Raman pulse shapes at the maximum pump power when FBG2 and FBG3 are added.

Furthermore, we measured the shapes of the output high-order pulses during the cascaded Raman conversion in Setup 2. As a result of the weakness and the absence of the lines, we only obtain the result when the width of the pump pulse is 20 ns and the repetition frequency is adjusted to 3 MHz, as shown in the following figure. Due to the good reflection ability of these FBGs at 1645 nm, almost all of the first-order Raman laser is reflected into the gas cavity and interacts with the deuterium continuously, which promotes the cascaded Raman conversion. We only detected the Raman pulse at 1695 nm at the forward output when the pump power is the maximum, and its shape is shown in Figure 4a. It can be seen that the pulse width is much narrower than 20 ns. In fact, there is another second-order Raman line at 1730 nm, as shown in Figure 2a, but, due to the low transmittance of the bandpass filter at this wavelength and its own instability, we cannot detect this pulse. We will discuss its characteristics in Section 3.3. Figure 4b presents the shape of the backward Raman pulse at 1695 nm, and it has a similar shape to that of the forward Raman pulse but is much weaker. The protuberance besides the pulse is the disturbance caused by the detector.

### 3.3. Output Power Characteristics

**Figure 5:**
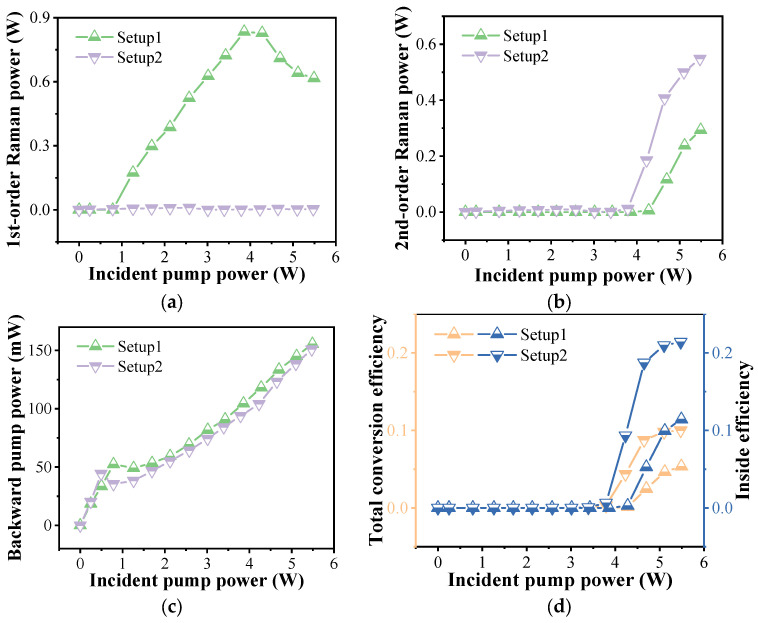
The characteristics of the output power in the different experiment setups: (**a**) forward Raman power, (**b**) backward Raman power, (**c**) backward pump power, and (**d**) the corresponding conversion efficiency.

When the pulse width is set to 20 ns and the repetition frequency is 3 MHz, we measured the power characteristics of the FGRL with a different experiment setup, as shown in Figure 5. For the first-order Raman power in Figure 5a, as the increasing of the incident pump power in Setup 1, we can obtain the maximum first-order Raman power of ~0.8 W when the incident pump power is ~3.9 W, and the corresponding total conversion efficiency is ~21.6%. Subsequently, the output Raman power decreases gradually, which is mainly caused by the cascaded Raman conversion. In Setup 2, although the cascaded Raman conversion has occurred inside the gas cavity (as discussed early in Section 3.1), we still cannot detect the Raman power. The reason is that the laser at 1645 nm is reflected by the FBG3, and the second-order Raman laser at 1765 nm is too weak to be measured. In addition, there is no pump power measured, which is consistent with the results in Figure 2. 

Then, we measured the cascaded second-order Raman power, and the curves of the power are shown in Figure 5b. It is clear that both curves show a similar trend, and, when the incident pump power exceeds the cascaded Raman threshold power, the second-order Raman power increases almost linearly. For Setup 1, the average threshold power of the cascaded Raman laser is ~4.3 W and the maximum cascaded Raman power of ~0.3 W is obtained when the pump is at the maximum. When the FBG3 in Setup 2 is introduced, the cascaded Raman conversion is enhanced greatly. The average power of the cascaded Raman threshold is reduced to ~3.8 W, meaning an 11.4% reduction in peak power. The maximum cascaded Raman power is also increased to ~0.5 W, which is 1.9 times the power in Setup 1. 

The curves of the backward pump power in Figure 5c increase at first, which is mainly due to the increasing incident pump power reflected by the FBG1. When the incident pump power exceeds the average power of the Raman threshold, the first-order Raman conversion occurs. It can be seen that the introduction of FBG3 at the Raman wavelength can reduce the threshold power, which has been demonstrated in our previous work [36]. Subsequently, the residual pump power reduces gradually, causing the decrease in the curves, and the backward pump power is mainly from the Fresnel reflection of splice 1. As a result of the same pump source in the two setups, the two curves are almost consistent with each other. 

We have also calculated the second-order Raman conversion efficiency of the cascaded FGRL, as shown in Figure 5d. For Setup 1, the maximum total conversion efficiency of 5.34% is obtained when the incident pump power is the maximum. But, when the splicing loss of splice 1 and splice 2 is considered, the corresponding efficiency inside the gas cavity is 11.4%. For Setup 2, due to the promotion of the FBG3, the total conversion efficiency is improved to 10.0%, and the inside efficiency is 21.4%, which is higher than that of the reported cascaded FGRLs [20,21,29]. By optimizing the splice loss and the fiber length, this efficiency is expected to be further improved.

**Figure 6:**
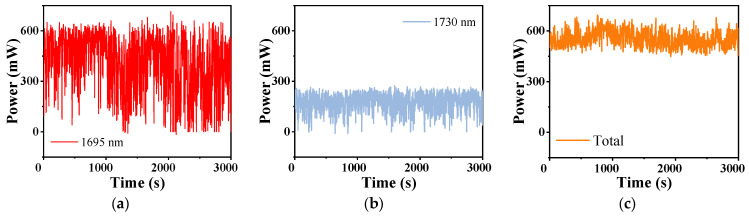
The power stability within 3000 s of the cascaded laser at (**a**) 1695 nm, (**b**) 1730 nm, and (**c**) the total of them.

As a matter of fact, for the cascaded Raman laser in Setup 2, there are two components at 1693 nm and 1730 nm. We separated them by two bandpass filters and measured their power stability successively when the pulse width was 20 ns and the repetition frequency was 3 MHz, as shown in Figure 6. It is noted that the stability of the total cascaded Raman power in Figure 6c is measured without using the filter. We can see that all of them are volatile and fluctuate greatly. For better comparisons, the data are analyzed by calculating, as shown in Table 1. Apparently, both the powers of the laser at 1695 nm and 1730 nm have a larger fluctuation range than that of the total power. We calculated the average power of them in 3000 s and the total average power is almost the sum of that of the two separated lasers. The standard deviation reflects the fluctuation degree of power. The fluctuation of power at 1695 nm is the largest, but the fluctuation of total power is relatively small. Thus, we only measured the total power as shown above to present the power evolution of the cascaded Raman laser.

**Figure 7:**
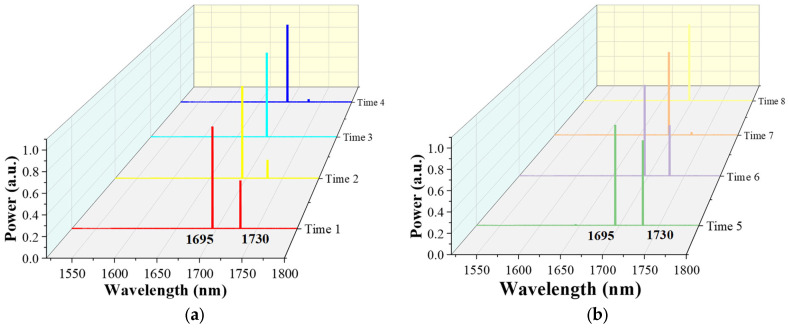
The output spectra of the cascaded FGRL in different times (**a**,**b**).

Furthermore, in order to explore the relationship between the cascaded lasers at 1695 nm and 1730 nm, we measured the output spectrum of the cascaded lasers at different times by the optical spectrum analyzer with a high resolution and plotted the results in the linear co-ordinate system showing in Figure 7, which is more intuitive to show the variations. As we can see, the power of the 1695 nm laser dominates, while that of the laser at 1730 nm changes randomly. We think this is mainly caused by the competition between the two. The laser at 1695 nm with a Raman shift of ~179 cm^−1^ has a lower Raman gain but its corresponding transmission loss is also lower, while the laser at 1730 nm with a Raman shift of ~298 cm^−1^ has a much higher Raman gain [40], but is much easier to be depleted due to the higher transmission loss. Therefore, when the coupled pump power remains constant, this competition leads to fluctuations in the cascaded Raman power. Since competition causes output power fluctuations, which affects the stability and reliability of the FGRLs, the competition should be suppressed as much as possible in practical applications. On the one hand, referring to various dopants or different materials in the solid-core fiber to change the Raman gain [41,42,43], a special gas-mixing system could be designed to control the distribution and concentration of gas components to reduce gain competition. On the other hand, spectral filtering techniques can be used to select the output wavelength. For example, FBG can be used to selectively enhance the desired cascaded Raman lines and suppress unwanted cascaded Raman lines.

## 4. Conclusions

We have demonstrated an all-fiber gas Raman laser oscillator at 1.7 μm based on the D_2_-filled HC-PCFs. An all-fiber gas cavity is fabricated by a 49 m HC-PCF filled with ~24.5 bar of deuterium and solid-core fibers, and a high-reflectivity FBG at 1540 nm is introduced at the output end. When pumped by the EDFA, the 1645 nm first-order Raman laser is obtained with a maximum power of ~0.8 W. An all-fiber cascaded gas Raman laser oscillator is realized by introducing FBGs beside the cavity. The maximum cascaded Raman power is increased by 1.8 times and the Raman threshold is reduced dramatically. The competition relationship between the second-order Raman laser at 1695 nm and 1730 nm is also studied. By optimizing the splicing loss and fiber length, the output power and conversion efficiency can be further improved. This work is a meaningful exploration of all-fiber cascaded FGRL, which is beneficial for realizing the laser output with an unobtainable wavelength.

## Figures and Tables

**Table 1:** The comparison of the power stability.

Characteristics	1695 nm	1730 nm	Total
Fluctuation range (W)	0–0.71	0–0.27	0.45–0.69
Average power (W)	0.40	0.17	0.55
Standard deviation (W)	0.1648	0.0503	0.0446

## Data Availability

The data presented in this study are available upon request from the corresponding author. The data are not publicly available due to privacy.
